# The Foreign Journals: Anatomy and Physiology

**Published:** 1841-01

**Authors:** 


### PART THIRD.

### Selections from the British and Foreign Journals.

### I. THE FOREIGN JOURNALS.

### ANATOMY AND PHYSIOLOGY.

*Memoir on the Relations which exist between Blood, Pus, Mucus, and Epidermis.* By L. Mandl, M.D.

This is a long memoir read before the *Société Médicale d'Emulation*, on June 3, 1840. We have only space for the general conclusions of the author; they are as follows: 1. The fibrinous globules of the blood, the globules of mucus, and those of pus, are identical. 2. All the globules are the product of the coagulation of the fibrine in the serum, which has transuded through the walls of the blood-vessels. 3. The liquid in which the globules swim constitutes the difference between pus and mucus. 4. If the fibrinous globules remain fixed to the surface of the membrane where they are secreted, they become the nuclei of epidermoid cellules, which constitute the elements of the epidermis. 5. If, on the contrary, the fibrinous globules remain free on the surface of the membrane, they are expelled by the organism, and form an element of pus and mucus. 6. These two liquids are simply filtered blood; that is to say, they contain all the elements of the blood, except the globules: the serum at the same time undergoing chemical alterations.

*Gazette Médicale de Paris. Juillet,* 1840.

*Menstruation in a Child.* By Dr. Lens, of Danzig.

A healthy woman, the mother of several hearty children, was delivered two years ago of a well-formed girl, which she, as usual, suckled herself. In the course of the year the child was weaned, and in the second year continued growing; but its parents were not a little surprised, when, in its eighteenth month, a flow of blood from the genitals took place, continued for some days, and then ceased. When I saw the child it was two years old and perfectly healthy, although the discharge of blood had, since the time mentioned, recurred regularly at every new moon. The breasts and genital organs were just like those of other children of the same age and sex; but the parents stated that the latter were hotter than usual, and somewhat swollen, before and during the discharge of blood, and that at the same time the child was always ill, with disturbance of the vascular system and heat about the head, great thirst, redness of the face, loss of appetite, and so on; and that when the discharge ceased, the child was always immediately after restored to its good health. The flow of blood always continued for three or four days, and, according to the parents' account, amounted to two, or, at the most, three ounces each time. As the discharge did not appear to interfere permanently or seriously with the child's health, the author did not attempt to control it.

*Casper's Wochenschrift. October* 3, 1840.

### *On the natural Existence of Copper and of Lead in the Human Body.*
### By Professor F. de Cattanei di Momo, of Pavia.

Having conceived the idea that the copper and lead, stated by Devergie to exist naturally in the human tissues, might have been received into the body with the food and from other sources, the author instituted experiments to determine whether those metals existed in the bodies of children that died a few days after birth, and had taken nothing but their mother's milk.

He took, therefore, 1st, the stomach and small intestines of a seven-months' child that had lived two days, and had taken only a little milk; 2d, the lungs, heart, and whole intestinal canal of another seven-months' child, fed, and having died in the same way; 3d, the lungs, digestive canal, liver, and spleen of a child born at the full term, and that had lived on its mother's milk for twenty-five days. The digestive canals of all three, the hearts and lungs of the first two, and the liver and spleen of the third were carbonized separately in Hessian crucibles, to prevent any suspicion that copper might be extracted from some alloy of silver if vessels of that metal were used, and that lead might proceed from the varnish if porcelain vessels were employed. The animal matters being thoroughly charred they were incinerated, and to accelerate the operation, and for the sake of contrast, nitric acid was used with one product and chlorate of potash with the other. Acetic acid was then poured on the ashes, and its action assisted by heat; an excess of ammonia was then poured into the solution, and, after standing for a time, the clear liquid was poured off. It had no azure tint, nor was any precipitate obtained from it by the double yellow cyanuret of potassium, or by hydrosulphuric acid, or by the immersion in it of a piece of polished iron. The solid portion, obtained by the addition of the excess of ammonia, was then redissolved in acetic acid, and, after filtration, there were added successively to portions of the clear fluid iodide of potassium, chromate of potash, and hydrosulphuric acid; and a piece of zinc was placed in it. The only reagent that could excite any suspicion of the existence of lead was the hydrosulphuric acid, which made the liquid slightly brown. The deposit that subsequently formed was redissolved in very dilute hydrochloric acid, and yellow cyanuret of iron and potassium being added to the solution, produced a precipitate of an azure blue colour; showing that the brown colour before seen had depended on the existence of sulphuret of iron.

From these analyses it was concluded that not a trace of copper or of lead had been obtained from the viscera. The author was proceeding to experiments on portions of adult bodies, when he learnt that a commission of the French Academy of Medicine had been unable to confirm the opinion of M. Devergie respecting the existence of copper in the body. The report of the commission did not allude to the occurrence of lead, but the author conceives that the results he obtained are sufficient proofs of its non-existence.

*Annali Universali di Medicina. Aprile,* 1840.

---

### *Some Researches on the specific Odour of the Blood.*
### By Dr. C. Taddei de Gravina.

The object of the experiments whose results are given in this paper, was to test the truth of the well-known principle laid down by Barruel, and more or less acknowledged by other chemists, that the blood of each individual exhales an odour closely resembling that of the cutaneous perspiration, and so peculiar that the species, and even the sex of any animal from whom a given quantity of blood has been drawn, may be determined by it.

The details of the previous investigations may be found in any extended work on medical jurisprudence; those of the present included examinations of the blood of the ox, cow, and very young calf, an old and a very young hare, the goat, sheep, hog, horse and mare, dog, man and woman, and numerous species of birds. The blood of each was subjected to the action of pure sulphuric acid, and, in the exhalations that were then given off, the peculiar odour was in every case perceived. The only cases in which the odour of one kind of blood could be confounded with that of another were those of the ox, cow, and sucking calf, and those of the hare and leveret; but in each of these cases the aromatic principle of the blood of the adult animal, though similar to, appeared stronger and more fragrant than that of the young, although the respective quantities of each, and of the sulphuric acid with which they were treated, were the same. Nor were the odours of the blood of a man and of a woman unlike each other, nor those of the horse's and mare's blood. The odour of the blood in each case was not different from that of the cutaneous exhalation of the several animals; and, in like manner, the blood of several cocks and hens, and of not a few pigeons subjected to the action of sulphuric acid, or left to putrefy, always exhaled a peculiar odour; in the former case reminding one of that perceived on entering a poultry-house, and in the latter of that of a dove-cote: odours which are also exhaled from the skin of the chest and under the wings of those birds. An odour, analogous to that of these parts of the skin, was given out under similar circumstances from the blood of thrushes, sparrows, linnets, goldfinches, woodcocks, and turkeys; and none of these had in its odour anything in common with that of the above-mentioned mammalia or of man, or of any of the species of birds. It follows, therefore, 1st. That it is true that the blood of every vertebrate animal has in it an odoriferous principle, identical in all the individuals of the same species, and similar to the odour of the cutaneous transpirations, or, more properly speaking, of that part of it which gives to each animal its characteristic smell. 2d. That the notion of those who pretend to recognize to which, among a number of individuals of the same species, a given portion of blood belongs, is false.

After obtaining these results, the author proceeded to the similar investigation of the blood of persons labouring under different diseases and under various other peculiar circumstances; but in none of these cases could he detect any corresponding differences in its odorous exhalation. From all these, therefore, his conclusion is, that neither the differences of age, of constitution, of temperament, of sex, of habit and customs, or of modes of living, nor diseases, medicines, or pregnancy, induce any change in the specific aroma of the human blood, but that it always preserves the same general odour; being only sometimes more fragrant and acid, or more garlicky and nauseous in some than in other persons.

*Annali Universali di Medicina. Febbrajo,* 1840.

---

*Extraordinary Enlargement of the Kidneys in the Fœtus, from the Development of Hydatids in their Substance.* By Dr. F. OESTERLEN, of Murrhardt, in Wurtemberg.

DR. OESTERLEN was called in January last to a patient in labour, whose delivery was retarded by the extraordinary size of the child's abdomen, but before his arrival the fœtus was expelled by the natural efforts.

The child, a female, was still-born; it appeared to have reached the full period; its weight was nine pounds, that of the placenta, which appeared healthy, two pounds. On opening the enormously large abdomen scarcely anything was to be seen but two large bodies, which were situated one at each side of the cavity, and had forced the liver up close under the diaphragm, so that only a small part of it was visible. The two bodies were ascertained to be the kidneys; they resembled each other exactly in size, structure, colour, and form; their shape was natural, and they were both divided into lobules, as is usual in the fœtus; they each weighed nine ounces, were about five inches and a half long, and four inches broad at their broadest part; they were smooth on their surface, of a red colour, in parts verging towards violet, and studded everywhere with little granules of a dark blue or gray colour. These little bodies were hydatids, which, on dividing each kidney, were seen to occupy the whole of its substance. The smallest hydatids presented a diameter of less than a quarter of a line, the largest of about two lines, but the average diameter was about a line. The smallest were near the surface, and they became larger towards the centre of the organ. Their form was spherical, and they were perfectly transparent.

The pelvis of each kidney was small, and contained a little clear watery fluid. The only remains of the true parenchyma of the organ consisted of a firm, fibrous, reddish tissue, which was by no means abundant. The kidneys were well supplied with blood. The bladder contained only a few drops of a perfectly limpid fluid. All the other organs in the body were natural.

*Neue Zeitschrift für Geburtskunde.* Band viii. s. 384.

---

*On the Microscopic Constituents of Milk.* By Professor NASSE, of Marburg.

AFTER a careful microscopic examination of milk from pregnant and suckling women, as well as from a cow and a bitch, and a comparison of his results with those of Donné and other preceding observers, the author says that the following may be enumerated as the microscopic constituents of the normal secretion of the mammary gland: 1. The smooth, homogeneous, transparent oil-globules, to which, in addition to the common milk-globules, belong also the fine, scarcely measurable particles and the larger drops of oil which swim on the top of the milk. 2. The cream-globules which are distinguished from the oil-globules by their opacity and their facette-like aspect. 3. The granulated yellow corpuscles. 4. The lamella of epithelium. 5. The more or less turbid medium, in which the four preceding kinds of corpuscles are suspended.

The first, the common milk-globules, are composed entirely of fatty matter, which dissolves completely and rapidly in ether. No membrane can be seen investing them. In the first nine days after delivery, the largest globules measure $\frac{1}{200}$ of a line in diameter, afterwards they are as much as $\frac{1}{100}$, but many are found of a much smaller size, and through all periods of lactation, the microscope, as well as other means of examination, show that the proportion of oil-globules in the milk varies greatly in different persons and under different circumstances.

In perfectly fresh warm woman's milk, no other globules than these are sometimes found. But as soon as the milk has stood for some time exposed to the air, other corpuscles are discernible in it which are distinguished from the preceding by a greater definiteness, a less degree of polish, and an appearance of facettes. In size they are nearly similar to the oil-globules, but if the milk be examined some time after it is drawn a number are found much larger; $\frac{1}{50}$ of a line or even more in diameter. They are not so easily soluble in ether as the common milk-globules; they do not break up in drying, but they become clearer; acetic acid and ammonia have no influence upon them; they diminish for a time when the milk is boiled, but they reappear gradually as it cools again; when left at rest they collect on the surface and form the cream; they easily stick together, and butter is formed when they are collected in one homogeneous mass. It is evident that they acquire their peculiar characters after they are drawn from the gland-ducts, for the author, as he watched them on the field of the microscope, could see individual globules which were originally clear, becoming on a sudden quite dark, and assuming the several characters of the cream-globules.

The yellow granulated corpuscles are almost peculiar to the colostrum; after the first few days from delivery they cease to occur in the milk, and they disappear from it earlier in those who have borne several children than in primiparæ. They are not all spherical; the majority are flat. Their diameter is at most from $\frac{1}{200}$ to $\frac{1}{100}$ of a line; some are found measuring $\frac{1}{50}$ in length and $\frac{1}{75}$ in breadth. They consist of small clear globules of fatty matter, which are connected together by a firm cement which is unalterable by either ammonia or concentrated acetic acid, or by boiling. When the milk is left at rest, these globules collect on its surface; and when they exist in considerable numbers render it unfit for making butter. The author believes that they are not, like the preceding globules, formed by the action of the air, but that they are produced by the secreting surface of the gland-ducts, and are analogous to the mucus-cells which are cast off from the surfaces of many mucous membranes, and to which they are in many respects very similar.

*Müller's Archiv*, 1840. Heft iii., p. 258.

---

*Experimental Researches relating to the Mode of Transmission of Rabies.* By M. BRESCHET.

AT the sitting of the Royal Academy of Sciences, September 21, 1840, M. Breschet brought forward proofs of the contagious nature of hydrophobia. Horror of liquids, and other hydrophobic symptoms, have been observed in man independently of contagion, but they essentially differ from the rabies of inoculation. The term hydrophobia is not applicable to animals, as in them the horror of water is not an essential symptom. Rabies is always produced by inoculation, and MM. Breschet and Magendie proved that it may be transmitted from man to the dog by inoculating a dog with the saliva of a hydrophobic patient. Thirty-eight hours afterwards the dog became furiously rabid; he bit several other dogs, and these became successively rabid. Some of these observations appear to show that the virus becomes enfeebled, or loses its deleterious properties in passing through several persons. The period of latency of the poison is about twenty or thirty days. Some of the dogs drank water with avidity. The virus is transmitted from carnivorous to herbivorous animals; in an ass bitten by a mad dog the disease was evidenced in its most intense degree. Two horses inoculated by the saliva of a dog had the disease less marked. The saliva of the ass above mentioned was put under the skin of several dogs and rabbits, and they all became rabid. Birds were killed by inoculation, but did not exhibit symptoms of rabies.

Is the blood altered in rabies? M. Breschet answers that he has several times transfused the blood of a rabid into the veins of a healthy dog, but has never determined the development of rabies in this manner. It appears that the saliva of the diseased animals alone affords the necessary conditions for the transmission of rabies. The saliva, or slaver, is really an altered fluid, a humour in a true morbid state; or the vehicle of a deleterious principle, of a true rabic virus newly secreted, but the nature of which is as yet completely unknown to us. Rabies then is a virulent contagious disease, and not the effect of a moral affection. *Archives Génèrales de Médecine, Octobre,* 1840.

---

*Bronchocele in a Fœtus of about Eight Months.* By Professor F. MONDINI.

THE subject of this case was born in a prison in which its mother was confined for a theft. It was a male child and born dead; the placenta and membranes were unnaturally large, and the liquor amnii very abundant, and it was evident that it had been alive to within a very short time of its birth.

At the anterior part of the neck there was a large tumour, a bronchocele. It occupied chiefly the left side of the neck, and was of an oval tuberculous form, and was here and there spotted with bright red. Commencing above at the margin of the lower eyelid, and hideously pushing up the nose and the mouth, it extended laterally, and protruding the lips, descended of an enormous size down to the lower apex of the sternum, constantly tending more to the left than to the right. It was longer than it was broad, measuring in one direction three inches and eight lines, and in the other three inches and a half. From a more minute anatomical examination, it appeared that this tumour consisted of a peculiar alteration of the cellular tissue interposed between the blood-vessels that form the network, and covering the network itself; the network in the thyroid gland, which, as Soemmering says, may be compared with the so-called rete mirabile, which is found on the carotid and the ophthalmic arteries of ruminants. *Novi Commentarii Acad. Scient. Instituti Bononiensis* v. iii., *and Annali Universali di Medicina. Ottobre,* 1839.

